# The Expulsion of Bile
*Summary of a Paper read at a Meeting of the Bristol Medico-Chirurgical Society on 11th April, 1934.


**Published:** 1934

**Authors:** R. J. Brocklehurst

**Affiliations:** Professor of Physiology in the University of Bristol


### THE EXPULSION OF BILE.*

BY

R. J. Brocklehurst, D.M.,

*Professor of Physiology in the University of Bristol.*

---

It is generally recognized that the principal factors responsible for the flow of bile into the intestine are the hepatic secretory pressure and the activity of the gall-bladder. In conjunction with these must be considered the resistance offered to the passage of bile by the structure and activity of the terminal portion of the common bile-duct and the neighbouring part of the duodenum.

### Hepatic Secretory Pressure.

This factor has been very fully investigated in dogs by, amongst others, McMaster and Elman,[1] who employed the method of " altercursive intubation " ; the cystic duct is ligated, and the common duct cut and doubly cannulated in such a way that the bile travels from the liver through a rubber tube passing through the abdominal wall and back through a similar tube into the lower part of the common duct ; outside the body the two tubes are joined by a T-piece, which enables the bile pressure to be measured on a manometer. After the taking of food the liver will secrete bile against a progressive resistance until the pressure reaches about 350 mm. of bile, when secretion ceases. The importance of this factor in

* Summary of a Paper read at a Meeting of the Bristol Medico-Chirurgical Society on 11th April, 1934.

bile expulsion is especially obvious in animals which do not possess a gall-bladder, *e.g.* horse, deer, rat.

### DUODENAL RESISTANCE.

This consists of the sphincter of Gage (Oddi), surrounding the end of the common duct, and the muscle of the duodenal wall through which the duct passes obliquely. The resistance, as determined in the dog by the method of altercursive intubation,[2] varies considerably. At its lowest, during digestion, the pressure necessary to force bile into the duodenum is about 80 to 100 mm. bile. It is at its highest when fasting, when it is at least 200 mm., and much higher values than this have been recorded; this fact, coupled with the more or less steady secretion of bile by the liver, is probably responsible for "starvation icterus."

The flow of bile from the papilla of Vater is intermittent, being almost always synchronous with duodenal motility. In general the flow occurs when the tone of the duodenal muscle is low, and is stopped when there is increased tonus in the neighbourhood of the papilla. The passage of a peristaltic wave down the duodenum may result in a spurt of bile from the papilla when the wave first reaches it, owing to a "milking" action of the duodenal muscle on the oblique intramural portion of the common duct; this certainly occurs in the dog, where this part of the duct is long and very oblique.[3]

Towards the end of digestion, as the duodenal resistance increases, less bile flows into the duodenum, a large part of it being diverted into the gall-bladder.

### GALL-BLADDER.

The position of the gall-bladder as a diverticulum of the main extrahepatic biliary channel is of particular importance in relation to the movement of the bile. There is overwhelming evidence that when the duodenal resistance is high enough to prevent any considerable flow of bile into the duodenum, *e.g.* in the post-digestive period, bile is side-tracked into the gall-bladder. As measured in the dog, the pressure in the ducts must be at least 60 mm. bile before the bladder commences to fill ;[1] and the greater the duodenal resistance the more bile will flow into the bladder. This process is favoured by the relative thinness and extensibility of the gall-bladder wall and by the considerable power possessed by its mucosa to absorb fluid from the bile. The capacity of the bladder for absorption of water is indicated in experiments with altercursive intubation without ligature of the cystic duct ; under these conditions the pressure in the dog's biliary system does not rise above 100 mm. bile during the post-digestive period, when little bile is flowing into the duodenum.

The gall-bladder, by filling when bile is not required for digestion and, as is well recognized, by concentrating its contents, thus acts as an ideal reservoir for the storage of the important dissolved constituents of the bile until they are needed during the next digestive period.

Although one or two people have expressed the opinion that bile which enters the gall-bladder does not leave it by the cystic duct under normal conditions, being completely absorbed,[4] this is not generally believed ; and there is no doubt that evacuation of its contents through the biliary ducts normally occurs, as is evidenced by the flow of concentrated " B " bile into the duodenum soon after food is taken. The method of cholecystography, moreover, shows a shadow of decreasing size during the digestion of a meal ; and dyes, sand, etc., placed in the gall-bladder in animals have been shown to leave it during digestion.

*Possible methods of evacuation.*—The gall-bladder possesses a definite muscle coat, which has been shown to undergo slow spontaneous rhythmic contractions.[5,6] By the method of "triple intubation" (altercursive intubation with, in addition, cannulation of the gall-bladder) McMaster and Elman[1] showed that the pressure in the dog's bladder during digestion may reach 250 mm. bile or more as a result of tonic contraction of its muscle. By cholecystography in man and animals contraction rings have been seen in the bladder wall, also the shifting of stones; and occasionally the gall-bladder contents thus visualized have been seen to pass momentarily into the hepatic ducts as well as down the common duct during the digestion of a fat meal, indicating a slightly higher pressure in the bladder than in the liver. By direct observation in animals many have seen contractions of the bladder wall; and in a few cases this has also been noted at laparotomy in human subjects when suitable materials have been placed in the duodenum by intubation, "B" bile being obtained by duodenal suction synchronously with the contractions.

Some have doubted the effectiveness of the relatively thin gall-bladder muscle to expel its contents by active contraction, believing that its function is rather to adapt itself by increasing tone to the progressive reduction of the bile in the bladder produced by, for example, the milking action of duodenal peristalsis on the lower part of the common duct. In experimental acute cholecystitis,[7] however, when the bladder muscle does not contract and duodenal movements are unaffected, evacuation of the bladder contents does not occur; and if the common duct is cut above the duodenum evacuation from a normal bladder still occurs with feeding of appropriate substances. There is, therefore, no doubt that the gall-bladder does actively expel its contents by muscular contraction under suitable conditions.

There is a possible accessory mechanism assisting the evacuation of the bladder bile. Graham and his colleagues[8] have emphasized the importance of the considerable amount of elastic tissue in the bladder wall, and have shown that the contents of a rubber balloon substituted for the gall-bladder are partially evacuated with similar time relationships to those of a normal gall-bladder. This is explained by the stretching of this elastic tissue by bile entering the bladder from the liver when the duodenal resistance is high, and by the subsequent recoil of the elastic fibres when the resistance falls, thus expelling bile into the common duct and duodenum. In fact, the elastic wall of an isolated gall-bladder behaves similarly to a rubber balloon when subjected to identical changes in internal pressure,[9] though it must be noted that only partial emptying of either ever occurs. This factor is almost certainly of slight importance as compared with the muscular contraction considered above, although it may play a part in the evacuation of the bladder contents early in digestion when the bladder itself is considerably distended.

Other suggested factors, which probably play no part (or at the most a very slight one) in the actual expulsion of bile, include siphonage at the junction of the cystic, hepatic and common ducts, gravity and changes of intra-abdominal pressure.

*Conditions of evacuation.*—The gall-bladder has been seen to evacuate in a variety of conditions, *e.g.* (1) moderate vagal stimulation ; (2) the use of parasympathomimetic drugs, of pituitrin and sometimes of histamine (when the common duct is not too tightly constricted by the duodenal muscle) ; (3) stimulation of the pyloric region and distension of the stomach ;

(4) in some animals the sight and smell of food (conditioned reflex), though this is said not to be effective if the resulting gastric juice is not allowed to enter the duodenum; (5) dilute hydrochloric acid or a strong solution of magnesium sulphate in the duodenum; (6) when food is eaten. Quite the most powerful gall-bladder evacuant is a meal of egg-yolk and cream.[10] Digested fats are far more effective than proteins and carbohydrates.

*Mechanism of gall-bladder contraction.*—Partial emptying of the bladder in man has been observed cholecystographically[11] as a result of chewing and ensalivating food, even without swallowing it. This must be a reflex effect, the efferent pathway being presumably the vagus. There is no doubt as a result of experiment in animals that reflex contraction of the gall-bladder muscle follows certain types of stimulation of the stomach and duodenum.[12]

But the completely denervated gall-bladder of the cat and dog expels its contents normally after a fat meal.[13] Therefore an extrinsic nervous mechanism is almost certainly not essential for normal evacuation, and we are driven to consider the possibility of a humoral mechanism. That such a mechanism exists is shown by the following observations.

The human bladder evacuates after the transfusion of blood from a donor who is digesting egg-yolk, but not after transfusion from a fasting donor;[14] and cross-circulation experiments in animals show that the presence of dilute hydrochloric acid in the duodenum of one animal evokes contraction of the gall-bladder of the other animal as well of its own. There is conflicting evidence as to the efficacy of absorbed food substances in the blood-stream producing a direct stimulation of the bladder muscle, and most workers in this field doubt this possibility. We are then left with the possibility of the production of a gall-bladder hormone, liberated into the blood as a result of the action of food substances, etc., on the wall of the small intestine.

It was shown by Brugsch and Horsters[15] that gall-bladder contraction could be induced by secretin, the hormone which arises from the small intestine and stimulates the production of pancreatic juice. This suggestion was reinvestigated by Ivy and Oldberg,[16] who found that intravenous injection of preparations of secretin extracted from the duodenum caused a rapid rise of gall-bladder pressure to about 240 mm. bile in dogs, which lasted from one-half to one hour. The administration of such extracts at ten minute intervals frequently emptied the gall-bladder within an hour. The response of the gall-bladder muscle is a general increase in tonus. Further, purification of the duodenal extracts showed that this active substance, though very similar to it chemically, was not identical with secretin and could be separated from it, and the name "cholecystokinin" was proposed. When this material is injected intravenously in animals and human subjects in very small doses gall-bladder contraction, and sometimes complete emptying, results.

### SUMMARY.

The existing state of our knowledge of the principal factors normally promoting bile expulsion can thus be briefly summarized.

The fundamental factor is the secretory pressure of the liver, modified by the resistance offered to the flow of bile by the degree of tone of the common duct sphincter and of the duodenal muscle. This factor is augmented by the active contraction of the muscle of the gall-bladder expelling its contents during digestion, and brought about partly by reflex stimulation, but chiefly by the action of a hormone liberated from the upper intestine when digested foods (principally fats), gastric acid, etc., pass into it from the stomach. To a slight extent this evacuation of the gall-bladder may be assisted early in digestion by the elastic recoil of the bladder wall when the duodenal resistance to the flow of bile in the common duct is lowered.

### REFERENCES.

[1] McMaster, P. D., and Elman, R., *Jour. Exper. Med.*, 1926, xliv. 173.

[2] Elman, R., and McMaster, P. D., *Jour. Exper. Med.*, 1926, xliv. 151.

[3] Burget, G. E., *Amer. Jour. Physiol.*, 1925, lxxiv. 583; 1926, lxxix. 130.

[4] Sweet, J. E., *Internat. Clin.*, 1924, i. 187; Halpert, B., *Med. Klin.*, 1924, xx. 408 and 1,830.

[5] Doyon, M., *Arch. Physiol. Norm. et Path.*, 1893, v. 678 and 710.

[6] Bainbridge, F. A., and Dale, H. H., *Jour. Physiol.*, 1905, xxxiii. 138.

[7] Murphy, G. T., and Higgins, G. M., *Arch. Surg.*, 1930, xx. 756.

[8] Graham, E. A., *Brit. Med. Jour.*, 1926, ii. 671; Graham, E. A., Cole, W. H., Copher, G. H., and Moore, S., *The Diseases of the Gall-Bladder and Bile Ducts*, 1928 (Philadelphia).

[9] Kodama, S., *Amer. Jour. Physiol.*, 1926, lxxvii. 385.

[10] Boyden, E. A., *Anat. Rec.*, 1923, xxiv. 388.

[11] Nemours-Auguste, *Presse Méd.*, 1933, ii. 1,106.

[12] Boyden, E. A., *Anat. Rec.*, 1928, xl. 147; Boyden, E. A., and Saunders, A. M., *Proc. Soc. Exper. Biol. and Med.*, 1928, xxv. 458; Birch, C. L., and Boyden, E. A., *Amer. Jour. Physiol.*, 1930, xcii. 301.

[13] Whitaker, L. R., *Jour. Amer. Med. Assoc.*, 1927, lxxxviii. 1,542.

[14] Sandblom, P., *Acta Radiol.*, 1933, xiv. 249.

[15] Brugsch, T., and Horsters, H., *Arch. exper. Path. u. Pharm.*, 1926, cxviii. 267.

[16] Ivy, A. C., and Oldberg, E., *Proc. Soc. Exper. Biol. and Med.*, 1927, xxv. 113; 1928, xxv. 251; and later papers.

The above is a brief selection of some of the work referred to. Very full lists of references on this subject can be found in Babkin, B. P., *Die Äussere Sekretion der Verdauungsdrüsen*, second edition, 1928 (Berlin), and in a recent review on the physiology of the gall-bladder by Ivy, A. C., *Physiol. Reviews*, 1934, xiv. 1.

